# Cytotoxicity Evaluation of Turmeric Extract Incorporated Oil-in-Water Nanoemulsion

**DOI:** 10.3390/ijms19010280

**Published:** 2018-01-17

**Authors:** Hee Jeong Yoon, Xiaowei Zhang, Min Gyeong Kang, Gyeong Jin Kim, Sun Young Shin, Sang Hong Baek, Bom Nae Lee, Su Jung Hong, Jun Tae Kim, Kwonho Hong, Hojae Bae

**Affiliations:** 1 College of Animal Bioscience and Technology, Department of Bioindustrial Technologies, Konkuk University, Hwayang-dong, Kwangjin-gu, Seoul 05029, Korea; rose_0013@naver.com (H.J.Y.); zhangxiaowei9304@gmail.com (X.Z.); vq1004pv@naver.com (M.G.K.); kkg0118@gmail.com (G.J.K.); 2 Laboratory of Cardiovascular Regeneration, Division of Cardiology, Seoul St. Mary’s Hospital, The Catholic University of Korea School of Medicine, Seoul 02841, Korea; tempast606@naver.com (S.Y.S.); whitesh@catholic.ac.kr (S.H.B.); 3 Department of Food Science and Technology, Keimyung University, Daegu 42601, Korea; bomnae1110@gmail.com (B.N.L.); tnwjd0799@naver.com (S.J.H.); jtkim92@kmu.ac.kr (J.T.K.); 4 KU Convergence Science and Technology Institute, Department of Stem Cell and Regenerative Biotechnology, Konkuk University, Hwayang-dong, Kwangjin-gu, Seoul 05029, Korea; hongkh27@gmail.com

**Keywords:** turmeric, curcumin, nanoemulsion, cytotoxicity

## Abstract

To overcome the drawbacks of conventional drug delivery system, nanoemulsion have been developed as an advanced form for improving the delivery of active ingredients. However, safety evaluation is crucial during the development stage before the commercialization. Therefore, the aim of this study was to evaluate the cytotoxicity of two types of newly developed nanoemulsions. Turmeric extract-loaded nanoemulsion powder-10.6 (TE-NEP-10.6, high content of artificial surfactant Tween 80), which forms the optimal nanoemulsion, and the TE-NEP-8.6 made by increasing the content of natural emulsifier (lecithin) to reduce the potential toxicity of nanoemulsion were cultured with various cells (NIH3T3, H9C2, HepG2, hCPC, and hEPC) and the changes of each cell were observed followed by nanoemulsion treatment. As a result, the two nanoemulsions (TE-NEP-10.6 and TE-NEP-8.6) did not show significant difference in cell viability. In the case of cell line (NIH3T3, H9C2, and HepG2), toxicity was not observed at an experimental concentration of less than 1 mg/mL, however, the cell survival rate decreased in a concentration dependent manner in the case of primary cultured cells. These results from our study can be used as a basic data to confirm the cell type dependent toxicity of nanoemulsion.

## 1. Introduction

Emulsions are small-sized droplets dispersed using two immiscible solutions with appropriate surfactants and are called nanoemulsion if the size of the droplet is at nano-scale. Since nanoemulsions can be fabricated in many different forms (liquids, creams, sprays, gels, aerosols, and foams), they can be employed in a variety of applications, including food and pharmaceuticals [1,2]. For instance, bioavailability can be significantly improved through the encapsulation of poorly soluble active ingredients, thus providing improved applicability [3,4]. 

Turmeric extract powder (TEP) used in this study for the production of curcumin nanoemulsion, is a mixture containing a number of commercially available vegetable supplements [5]. Among them, the pharmacological activity of curcumin, an index substance of turmeric, has been reported through many studies [6,7,8]. However, curcumin, a yellow hydrophobic polyphenolic component extracted from turmeric has limited applications due to poor solubility and toxicity at high concentrations [9,10,11]. Moreover, its low bioavailability in the body remains as a major issue. To solve these problems, curcumin nanoemulsion was prepared by using TEP to increase applicability and bioavailability of curcumin.

Although nanotechnology have emerged as a technology that could positively modify or control the functionality, stability, and usefulness of the active substances for high value-added products [12,13,14], the regulation of nanotechnology incorporation varies in different countries and methods as well as procedures for safety evaluation of nano-substances are also being implemented [15,16,17,18,19]. For example, the toxicity of the nano-sized materials cannot be completely identified in the human body, which is one of the major obstacle for commercialization (e.g., food, cosmetics or drugs) [20,21]. Therefore, the safety evaluation of the nano-materials is crucial.

**Figure 1:**
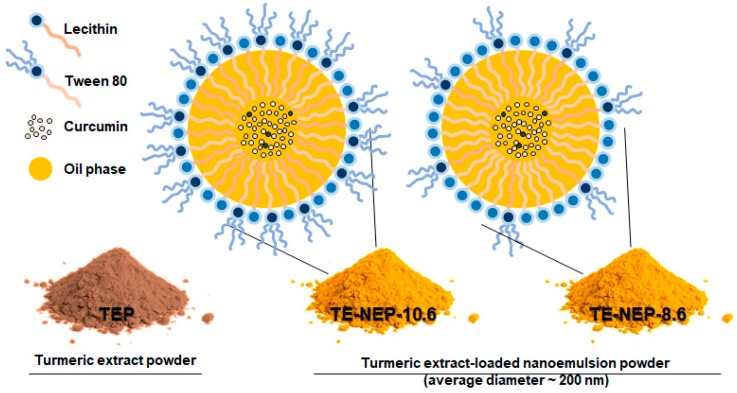
Schematic of o/w nanoemulsion including curcumin. Turmeric extract-loaded nanoemulsion powder (TE-NEP) was prepared using Turmeric extract powder (TEP). TE-NEP-10.6 has a HLB value of 10.6, and TE-NEP-8.6 has a HLB value of 8.6. Abbreviations: o/w, oil in water; HLB, hydrophilic lipophilic balance.

In this study, the toxicity of nanoemulsion as a carrier to improve the bioavailability of bioactive substances such as curcumin (extracted from TEP), and the possibility of its application has been verified through evaluating the potential toxicity. Two types of oil-in-water nanoemulsions containing curcumin were prepared as shown in Figure 1 and Table 1. TE-NEP-8.6 was produced by lowering the ratio of synthetic emulsifier (tween 80). The toxicity of each emulsion was evaluated at the cellular level (3-(4, 5-dimethylthiazol-2-yl) 2, 5-diphenyl tetrazolium bromide (MTT) assay, lactate dehydrogenase (LDH) assay, Live/dead assay, and comet assay).

## 2. Results

### Cytotoxicity of Turmeric Extract-Loaded Nanoemulsion

**Figure 2:**
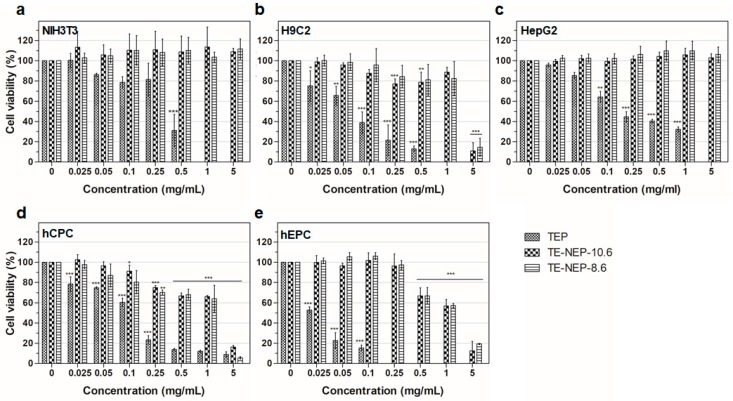
Evaluation of cell viability by MTT assay on (**a**) NIH3T3 cell line, (**b**) H9C2 cell line, (**c**) HepG2 cell line, (**d**) hCPC primary cell, (**e**) hEPC primary cell treated with Turmeric extract powder (TEP) and turmeric extract-loaded nanoemulsion powder with HLB values of 10.6 (TE-NEP-10.6) and 8.6 (TE-NEP-8.6), respectively. Cells were incubated with nanoemulsion samples (0.025, 0.05, 0.1, 0.25, 0.5, 1 and 5 mg/mL) for 24 h. Experiments were repeated 3 times independently. *, **, *** *p* < 0.05, compared to the control.

To assess the cell viability activity of turmeric extract-loaded nanoemulsion powder (TE-NEP) against various cell types (NIH3T3, H9C2, HepG2, hCPC, and hEPC, respectively), MTT assay was performed to evaluate the cell metabolic activity of living cells. Both cell lines and primary cells were incubated with different concentrations of TE-NEP, TE-NEP-10.6, and TE-NEP-8.6 (0–20 mg/mL). Cell viability was determined after 24 h of incubation. As can be seen in Figure 2, some of the extracts induced cell cytotoxicity in certain concentrations. In all cell types, TEP induced cytotoxicity in proportion to its concentration. In the case of cell lines, toxicity was not observed after the treatment with two nanoemulsions except for H9C2 at a concentration of 5 mg/mL (Figure 2a–c). The two nanoemulsion samples showed toxicity in proportion to the concentration of the sample at concentrations above 0.25 mg/mL for hCPC and above 0.5 mg/mL for hEPC (Figure 2d,e). The results of positive control are shown in Figure S2. Since the curcumin content varied between three groups (turmeric extract and two nanoemulsions), the MTT assay was performed by matching the content of curcumin to evaluate the toxicity according to its exact content. As a result, the toxicity of the TEP decreased (Figure S3). The NIH3T3 cell line showed cell viability of less than 85% at the concentrations of 3.248 and 16.24 μg/mL in TEP and two nanoemulsion samples, respectively (Figure S3a). On the other hand, the cell viability of H9C2 cells tended to decrease in proportion to the concentration of curcumin. At the concentration of 0.812 μg/mL, all of the sample groups showed cell viability below 85% (Figure S3b). In the case of human hepatocytes (HepG2), TE-NEP-10.6 and TE-NEP-8.6 showed cell viability of 66 ± 2.7% and 27 ± 3.7% at concentration of 32.48 μg/mL, respectively (Figure S3c). In contrast, human-derived primary cells showed dosage-dependent cytotoxicity in all groups (Figure S3d,e). Compared with the results obtained by adjusting the content of curcumin, the high toxicity of TEP when treated with the same concentration of powder is due to the high concentration of curcumin which is toxic to the cells.

**Figure 3:**
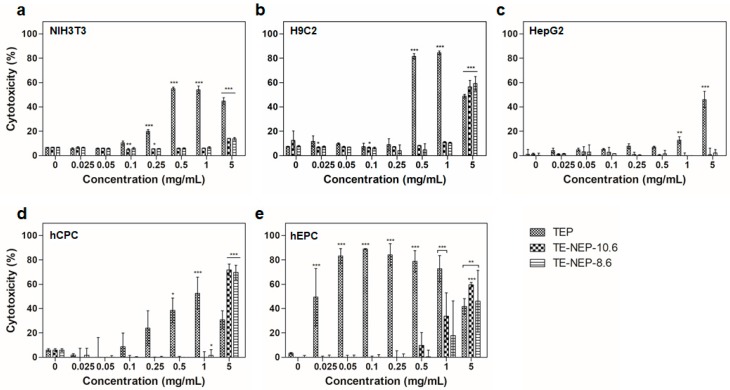
The cytotoxicity effects of TEP, TE-NEP-10.6 and TE-NEP-8.6 (0.025, 0.05, 0.1, 0.25, 0.5, 1 and 5 mg/mL) on (**a**) NIH3T3, (**b**) H9C3, (**c**) HepG2, (**d**) hCPC and (**e**) hEPC. Cell death was measured with the LDH assay after 24 h. Experiments were repeated 3 times independently. *, **, *** *p* < 0.05, compared to the control.

In addition, the cytotoxicity of the samples (TEP, TE-NEP-8.6, and TE-NEP-10.6) was assessed by LDH assay, which assessed cell damage by LDH released from damaged cells. In all cell lines, the LDH assay results of TEP and two nanoemulsion samples were similar to MTT assay results, but in HepG2, TEP showed toxicity at concentrations above 1 mg/mL (Figure 3a–c). Concentration dependent cytotoxicity was detected at hCPC treated TEP and the two nanoemulsions were toxic only at the highest concentration of 5 mg/mL (Figure 3d). On the other hand, hEPC showed high toxicity results regardless of concentration in TEP, and concentration-dependent toxicity was confirmed at higher than 0.5 mg/mL of two nanoemulsions (Figure 3d). Figure S4 shows the results of positive control according to each cell types. When the content of curcumin was matched, the LDH analysis results were similar to that of MTT assay (Figure S5). Overall, NIH3T3 and H9C2 showed high levels of cytotoxicity at 16.24 and 8.12 μg/mL, respectively (Figure S5a,b). In the case of HepG2, TEP showed a concentration-dependent cytotoxicity from 3.248 μg/mL, and the two nanoemulsions showed cytotoxicity at the highest concentration of 32.48 μg/mL (Figure S5c). For hEPC, the nanoemulsion showed concentration dependent cytotoxicity from 0.812 to 32.48 μg/mL, while for hCPC, the highest toxicity was observed at 8.12 μg/mL nanoemulsion concentration (Figure S5d,e).

**Figure 4:**
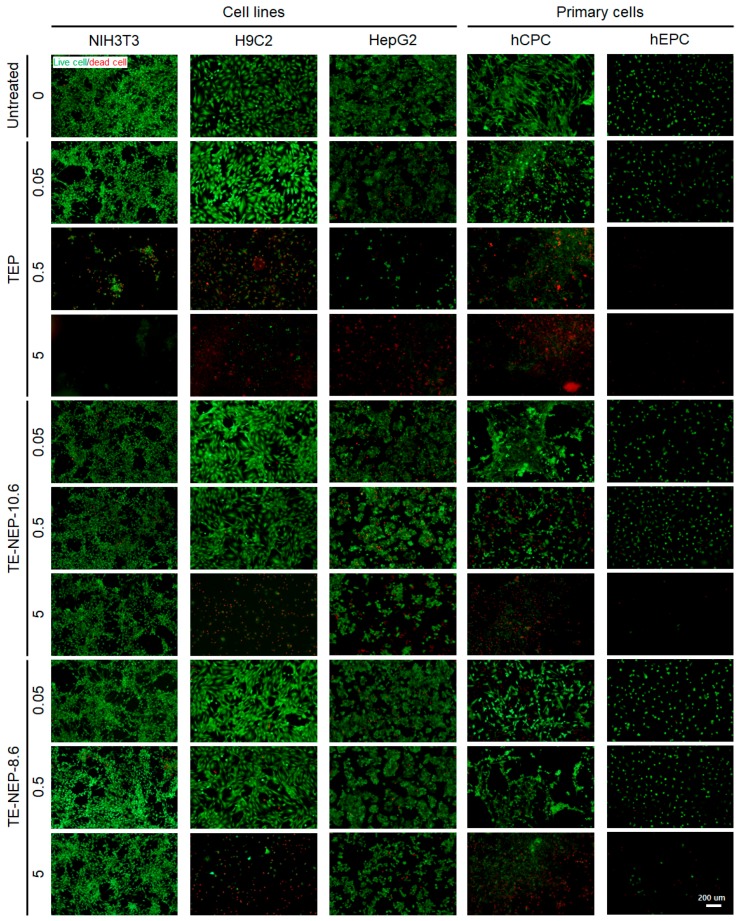
Representative fluorescence live/dead images of NIH3T3, H9C3, HepG2, hCPC, and hEPC. Each cell was stained with calcein-AM (green)/ethidium homodimer (red) LIVE/DEAD assay after the sample (TEP, TE-NEP10.6 and TE-NEP-8.6) treatment (24 h). Scale bar = 200 μm.

The viability of each cells was visualized by fluorescence staining (Figure 4). Live cells and dead cells were stained with calcein-AM and EthD-1, respectively. TEP was cytotoxic in a concentration-dependent manner in all cell types. The number of dead cells increased, and the viability decreased significantly at the highest concentration of 5 mg/mL. In NIH3T3 and HepG2, cells showed low toxicity against nanoemulsion. On the other hand, in the case of H9C2, it was confirmed that most of the cells were dead at 5 mg/mL. The primary cultured cells, hCPC, indicated definite concentration dependent cytotoxicity. hEPC showed significantly reduced cell density, similar to H9C2, due to the depletion of dead cells at a concentration of 5 mg/mL. Figure S6 implied quantification data for living cells. The live/dead test results for all experimental concentrations are shown in Figure S7.

## 3. Discussion

Mouse fibroblasts (NIH3T3), rat heart myoblasts (H9C2) were selected as representative animal cell line. Since the liver is a detoxifying organ where bulk of nutrients are received [22], HepG2 was chosen as representative of human-derived cell lines. Once received, metabolized nutrients are then released back into blood stream through the blood vessel, and blood is pumped throughout the body from the heart [23]. Therefore, human cardiac progenitor cells (hCPC) and human endothelial progenitor cells (hEPC) were selected as representative of primary human cells. In particular, it would be possible to evaluate more reliable toxicity towards humans by using various human-derived primary cells [24]. 

The TEP is a mixture containing a number of commercially available vegetable supplements [5]. Among them, the pharmacological activity of curcumin, an index substance of turmeric, has been reported through research [6,7,8]. Curcumin, a yellow hydrophobic polyphenolic component extracted from turmeric has limited applications due to low solubility and availability [9,10,11]. Moreover, its low bioavailability in the body remains as a major issue. To solve these problems, curcumin nanoemulsion was prepared by using TEP to increase applicability and bioavailability of curcumin. Nanoemulsions are widely applied in drug delivery systems and food applications due to their biocompatibility and permeability enhancing properties [25,26]. In addition, the materials used to make the nanoemulsion, water, oil (Medium-chain triglyceride (MCT) oil), and surfactant (Lecithin and Tween-80), have been approved for food use [27,28]. In particular, MCT oil is a vegetable oil, and lecithin is a component extracted from soybeans. According to the Material Safety Data Sheet (MSDS), both substances are highly safe food ingredients not classified as hazardous/hazardous substances. Tween 80 is a hydrophilic emulsifier and is the most widely used emulsifier to make oil in water nanoemulsion [29]. However, Tween 80 have been reported to be toxic in some cases and therefore the allowable amount of Tween 80 used as a food additive is 11.8 g/kg for nutritional supplement and 1 g/kg for non-standard processed food. The Tween 80 content of the nanoemulsion (TE-NEP) was 337.5 g/10 L, and after mixing with dextrin powder, the final Tween 80 contained in the dried powder (TE-NEP) was about 37 g/kg (3.7%) (Table 1). 

The content of curcumin, which is a target substance contained in the nanoemulsion, was confirmed by HPLC. The curcumin content of TEP was 32.48 μg/mL, and the curcumin contents of TE-NEP-10.6 and TE-NEP-8.6 were 1.83 and 1.64 μg/mL, respectively (Table 2). TEP has about 17 times higher curcumin content than nanoemulsion. Since curcumin can induce cell death according to its concentration, as well as exhibit various pharmacological activities [30] (e.g., antioxidation [27,31,32], antiinflammation [33,34], and antitumor effects [35,36,37]), our results showing cytotoxicity at high TEP with a high curcumin content can be explained. Furthermore, based on the No Observed Adverse Effect Level (NOAEL) value of curcumin (250–320 mg/kg bw/day), international expert scientific committee JECFA (joint FAO/WHO Expert Committee on Food Additives) have proposed an acceptable daily intake (ADI) of 3 mg/kg bw/day for insoluble curcumin. [38]. 

Applied nanoemulsions can potentially induce toxicity which may be caused by their small size [20]. For example, nanoparticles can penetrate the body through various pathways and remain in the system because they can escape phagocytosis through macrophages, as opposed to micro-sized particles. These residual nanoparticles with artificially created new chemical and physical properties can cause toxicity due to the ongoing body reaction and active state [39,40,41]. Also, due to their physico-chemical properties, such as their composition and concentration, particle size distribution, electrical properties, and interfacial properties, toxicities may change in nano-sized materials even if they were proven not to be toxic in micro- or macro-scales [20,42]. In addition, the toxicity test was performed by matching the curcumin content of TEP with the two types of nanoemulsion samples (TE-NEP-10.6 and TE-NEP-8.6), and cytotoxicity of the nanomaterial was confirmed. The nanoemulsion (TE-NEP-10.6 and TE-NEP-8.6) used in this study showed an average diameter of 196.93 ± 4.80 nm and 202.11 ± 4.21, respectively (Figure S1). The structure of nanoemulsion was confirmed by SEM analysis. There are three reasons why emulsion sizes are observed to be smaller in representative SEM images: (1) Particle size measured using dynamic light scattering technology can be changed by the angle of light reflection or the mobility of the nanoemulsion in the solvent [43,44]. (2) The dispersed emulsion can easily aggregate with one another and the size of the emulsion can be seen to be larger when measured in the coagulated state [44,45,46]. Finally, (3) the sample is dried during sample preparation for SEM analysis, at which time the size of the emulsion can be reduced. 

Since DNA damage is one of the important factors in tumor formation, there have been concerns regarding potential genotoxicity of the substance aimed for food or drug applications [47,48]. Previous research reports have shown that nanomaterials can cause direct or indirect DNA damage [49,50]. For instance, it has been reported that curcumin itself may cause DNA damage induced by oxidative stress at high doses (>8 μg/mL) in HepG2 cell lines [38,51]. These previous results support the genetic toxicity results induced by TE-NEP in this study. In our study, genotoxic effects were determined by comet assay to evaluate DNA damage of cells (NIH3T3, H9C2 and hCPC). This assay allows DNA damage quantification in highly efficient manner with good sensitivity. In particular, hCPC and hEPC showed higher cytotoxicity according to MTT, LDH and live/dead assay when comparing the results from the cell lines (Figure 2). Specifically, the comet assay results of hCPC showed significant differences in tail DNA% at all experimental concentrations (Figure S8b). This can be due to the fact that various cells have different characteristics such as growth environment, proliferation, and membrane properties. In general, primary cultured cells require stringent conditions such as various growth factors for cell culture and are difficult to survive in normal environments required for cell lines [24,52]. Tumor cells are also more likely to survive in harsh environments that can be caused by nanomaterials, due to their proliferative properties [53]. In addition, these cell specificities can explain relatively low cytotoxicity at several concentrations of HepG2, a tumor cell line, identified in MTT (Figure 2c) and LDH (Figure 3c) assays. Several studies also confirmed that the cytotoxicity of nanomaterials was relatively lower in the case of HepG2 compared to other cell lines [54,55,56]. In this study, the cellular toxicity of the prepared nanoemulsion was evaluated. Genetic toxicity assessment using comet assay was also performed. However, in the case of genotoxicity, further experiments such as micronucleus assay and gH2AX staining are required to produce more reasonable result. Ultimately, for practical applications in food and drug delivery systems, animal-level toxicity testing should be essential before the final application. 

## 4. Materials and Methods

### 4.1. Sample Preparation

Turmeric extract powder (TEP) was provided by Ottogi Co., Ltd. (Anyang, Gyeonggi-do, Korea). Two types of turmeric extract-loaded nanoemulsion powder (TE-NEP-8.6 and TE-NEP-10.6) were provided by J.T. Kim (Keimyung University, Daegu, Korea). Briefly, oil phase was prepared by dissolving 20% (*w*/*w* based on MCT) TEP in MCT oil containing soy lecithin. The aqueous phase was prepared by mixing tween 80 and distilled water. The amount of soy lecithin and tween 80 were adjusted to the hydrophilic lipophilic balance (HLB) values of 10.6 and 8.6. The coarse emulsion was prepared by magnetic stirring under ambient temperature for 2 h. Then, nanoemulsions were prepared by further homogenizing the coarse emulsion through high speed homogenization (HSH) (HG-15D, Daihan Scientific Co., Ltd., Wonju, Korea) at 5000 rpm for 10 min, ultrasonication (US) with a Vibra Cell (VCX-750, Sonics & Materials, Inc., Sandy Hook, CT, USA) for 15 min, and high-pressure homogenization under 10,000 psi for 3 cycles. TE-NEP-10.6 and TE-NEP-8.6 indicate the turmeric extract-loaded nanoemulsion with HLB values of 10.6 and 8.6, respectively. Control NE was prepared by using the same method without turmeric extract powder. TE-NEP was prepared by spray drying (KL-8, Seo Gang Engineering Co., Ltd., Cheonan, Korea) with TE-NE solution and dextrin. The spray drying conditions were as follows: inlet temperature at 120 °C, flow rate at 50 mL/min, and outlet temperature at 180 °C.

### 4.2. Cell Culture

#### 4.2.1. Cell Lines

Mouse fibroblasts (NIH3T3), rat heart myoblasts (H9C2), and human hepatoblastoma (HepG2) cells were purchased from Korean Cell Line Bank (KCLB, Seoul, Korea). Cells were cultured in high-glucose Dulbecco’s Modified Eagle’s Medium (DMEM; Welgene, Daegu, Korea) supplemented with 10% fetal bovine serum (FBS, Welgene) and 1X penicillin/streptomycin (P/S, Welgene). Cells were routinely incubated under humidified atmosphere containing 5% CO_2_ at 37 °C and subcultured when 85% confluent.

#### 4.2.2. Primary Cells

Human endothelial progenitor cells (hEPCs) and Human cardiac progenitor cells (hCPCs) were kindly provided by S.M. Kwon (Pusan University, Pusan, Korea, IRB number: 05-2015-133). The hEPCs were cultured on 1% gelatin coated dishes in EC basal medium 2 (EBM-2MV, Lonza, Walkersville, MD, USA) supplemented with 5% FBS, EGM-2-MV BulletKit and 1X P/S. The hCPCs were cultured in Ham’s F12 medium (Hyclone, Logan, UT, USA) containing 10% FBS, 1X P/S, 0.005 U/mL human erythropoietin (hEPO, R&D system, Minneapolis, MN, USA), 5 ng/mL human basic fibroblast growth factor (hbFGF, PeproTech, Rocky Hill, NJ, USA) and 0.2 mM L-glutathione reduced (Sigma-Aldrich, St. Louis, MO, USA). All of the cells were cultured under standard cell culture condition using 5% CO_2_ incubator at 37 °C and subcultured when 85% confluent.

### 4.3. Cytotoxicity Assay

#### 4.3.1. MTT Assay

The cytotoxicity of cells was observed by using 3-(4,5-Dimethylthiazol-2-yl)-2,5-diphenyltetrazoliumbromide (MTT, Duchefa, Haarlem, The Netherlands) assay. Briefly, cells were seeded on a 96-well plate and cultivated for 24 h at 37 °C (5% CO_2_). The appropriate cell density was selected according to the cell type. HepG2 at a cell density of 2 × 10^4^ cells, all other cells at 1 × 10^4^ cells per 100 μL medium were seeded into each well of the 96-well plates. Afterwards, the cells were exposed to the TEP, TE-NEP-10.6, TE-NEP-8.6 at the several concentrations (0–20 mg/mL), respectively and incubated for 1 day. Negative control was prepared by adding single-wall carbon nanotube (SWCNT) at concentrations of 0.025, 0.05, 0.1, 0.25, 0.5, 1, and 5 mg/mL. After the exposure, medium was changed followed by the addition of 5 μL of MTT reagent (5 mg/mL stock). The cells were incubated for 3 h at cell culture condition, and lysed in DMSO (100 μL per well). The development of color was measured spectrophotometrically using Epoch microplate spectrophotometer (BioTek Instruments, Winooski, VT, USA) at 570 nm. All absorbance values were corrected against blank wells. The cell viability was calculated by the following formula (*A* = absorbance):

$$\mathrm{Cell}\;\mathrm{viability}\;\left(\%\right)=\;\frac{A_{sample}-A_{blank}}{A_{control}-A_{blank}}\times 100$$

#### 4.3.2. Live/Dead Assay

A LIVE/DEAD Viability/Cytotoxicity Kit for mammalian cells (Thermo Fisher Scientific, Walthan, MA, USA) was used according to the manufacturer’s instructions to visualize cell viability. Briefly, 20 μL of 2 mM ethidium homodimer-1 (EthD-1) stock solution and 5 μL of 4 mM calcein-AM solutions were diluted in 10 mL of sterile and prewarmed Dulbecco’s Phosphate-Buffered Saline (DPBS, Welgene). The mixture was then mixed and distributed to cells in 96-well plates, followed by incubation for 45 min at room temperature. After the staining procedure, stained cells were imaged using fluorescent microscope (Nikon, ECLIPSE Ts2, Tokyo, Japan).

#### 4.3.3. Lactate Dehydrogenase (LDH) Assay

Lactate dehydrogenase (LDH) leakage into the culture medium from dead cells was measured using EZ-LDH Cell Cytotoxicity Assay Kit (Daeil Lab Service, Seoul, Korea). The LDH release assay was used according to the manufacturer’s direction. In brief, cells were seeded in 96-well plates. Optimal cell density was selected for each cell through the cell optimization step. Cells were treated with a range of concentrations (0–20 mg/mL) of each sample (TEP, TE-NEP-8.6 and TE-NEP-10.6) for 24 h. CNT was used a positive control and group without cells were prepared as a blank. After the incubation with treated sample for 1 day, the cultures were centrifuged at 600 g for 5 min. The high control, which was the maximum amount of LDH that could be released from a cell by artificially killing the cells, was combined with the lysis buffer before the collection of the supernatant and reacted for 5 min at room temperature. Following the centrifugation, 10 μL of the supernatant was transferred to new 96-well plate and 100 μL of LDH reaction mixture was added to each well and mixed carefully. The mixture was reacted at room temperature for 30 min in dark. Absorbance was measured at 450 nm using the microplate spectrophotometer after shaking gently. The percentage of total cellular LDH released was determined using the following equation:

Cytotoxicity = (A − B)/(C − B) × 100

where A indicates the OD value of Experimental LDH release, B is OD value of spontaneous LDH release, and C stands for OD value of maximal LDH release (high control).

### 4.4. Statistical Analysis

The statistical evaluations of results were analyzed by GraphPad Prism 5.0 (GraphPad Prism Software, Inc., San Diego, CA, USA). The values are expressed as mean ± standard deviation (SD). For comparison of multiple groups, one-way analysis of variance (ANOVA) with a post-hoc Bonferroni test was applied. For all analyses, *p* < 0.05 was considered as statistically significant. The significance of differences between two groups was performed by unpaired two-tailed Student *t* test.

## 5. Conclusions

Cell-level toxicity studies (MTT, LDH, Live/dead assay) were conducted for applications in food or drug delivery of nanoemulsions. Overall, the toxicity of the nanoemulsion did not significantly affect its composition and increased in a concentration-dependent manner. Results obtained from this study provide a basic knowledge of the cytotoxicity of nanoemulsion on various cell types and can be used as a basis for future animal experiments.

## Figures and Tables

**Table 1:** Composition of turmeric extract-loaded nanoemulsion powder (TE-NEP).

Sample	HLB Value	MCT Oil (g)	Surfactant (g)		DW (mL)	TEP (g)	Dextrin (g)
			Lecithin	Tween 80			
TE-NEP-8.6	8.6	500	400	100	9000	107.41	1107.41
TE-NEP-10.6	10.6	750	412.5	337.5	8500	161.11	1661.11

Abbreviations: HLB, hydrophile-lipophile balance; MCT, Medium-chain triglyceride; DW, distilled water; TEP, turmeric extract powder, TE-NEP-8.6, turmeric extract-loaded nanoemulsion powder with HLB value of 8.6; TE-NEP-10.6, turmeric extract-loaded nanoemulsion powder with HLB value of 10.6.

**Table 2:** Content of curcumin in TE-NEP.

Sample	AVE (μg/mL)	STD
TE-NEP-8.6	1.64	0.01
TE-NEP-10.6	1.83	0.02
TEP	32.48	0.46

AVE: Average; STD: Standard deviation.

## Supplementary Materials

The following are available online at www.mdpi.com/1422-0067/19/1/280/s1.
