# Supplementary material for: Cytotoxicity Evaluation of Turmeric Extract Incorporated Oil-in-Water Nanoemulsion
Source: Int J Mol Sci. 2018 Jan 17;19(1):280. doi: 10.3390/ijms19010280 (PMC5796226; doi:10.3390/ijms19010280)
Supplement: Supplementary file 1 [file ijms-19-00280-s001.pdf]

International Journal of *Molecular Sciences*

Supporting Information

1. Methods

Curcumin content in the TEP and TE-NEPs

Curcumin content of the turmeric extract powder (TEP), TE-NEP-10.6, and TE-NEP-8.6 was measured using a HPLC (Shimadzu D-20A HPLC, Shimadzu, Kyoto, Japan) fitted with an UV absorbance detector (operated at 265 nm) and an ACE5 C18 column (4.6 x 250 mm, 5 μm; Advanced Chromatography Technologies, Aberdeen, UK). The mobile phase was 100% methanol pumped at a flow rate of 1.0 mL/min. A sample volume of 20 μL was injected.

Characterization of nanoemulsion

The particle size and distribution of nanoemulsions (TE-NEP-10.6 and TE-NEP-8.6) were determined with dynamic light scattering (DLS) technique (NanoBrook Omni Particle Sizer and Zeta Potential Analyzer, Brookhaven Instruments Corporation, USA). The nanoemulsion suspension was diluted to concentration of 5 mg/mL with PBS. The morphology of the nanoemulsion (TE-NEP-10.6 and TE-NEP-8.6) were confirmed by field emission scanning electron microscopy (FE-SEM, Hitachi SU-8010, Japan). To prepare the sample, a double-sticky carbon tape was attached on the stub and 1-2 drops of the nanoemulsion dispersion and then completely dried. The samples were coated with gold and observed at an accelerating voltage of 15 kV using a gold sputter coater in a high-vacuum evaporator.

Genotoxicity

Genotoxicity was measured using an Alkaline Comet assay with slight modification of the protocol from the study by Céline Courilleau (2012) [1]. Briefly, the cells were treated with test samples (TEP, TE-NEP-10.6, and TE-NEP-8.6) at various concentrations (0-5 mg/mL) for 24 hr and then resuspended in 0.7% (*w*/*v*) low melting point agarose (LMP agarose, sigma) at 37 °C. Immediately, 20 uL of cell-LMP agarose mixture was spread onto a slide glass pre-coated with 0.85% (*w*/*v*) normal melting point agarose (Dongin Biotech, Seoul, Korea). The slides were incubated at 4 °C for 30 min, immersed in pre-chilled lysis solution (2.5 M NaCl, 100 mM EDTA, 10 mM Tris base, and 1% Triton X-100, pH 10) and incubated at 4 °C for 60 min in the dark. The slides were then placed in freshly prepared alkaline solution (300 mM NaOH, 1 mM EDTA, pH > 13), and electrophoresis was performed for 25 min at 300 mA and 25 V. After electrophoresis, the slides were fixed in 70% ethanol for 10 min, dried at room temperature and stored until staining step. To perform microscopic analysis, the slides were stained with Vista Green DNA dye (1:10,000 in TE buffer, Komabiotech, Seoul, Korea) for 10 min in the dark and imaged using a fluorescent microscopy at 200X magnification. The images were analyzed by using CASP v.1.2.2 software. At least 28 comets were analyzed per slide.

2. Results

Genotoxicity of Turmeric extract-loaded nanoemulsion

Comet assay was performed to evaluate whether the nanoemulsion containing curcumin caused DNA damage. Tail DNA% was used as a measure of DNA damage. NIH3T3 cells showed significantly higher tail DNA% in TE-NEP-10.6 samples at 0.025 and 0.05 mg/mL compared to the control group. Treatment of TE-NEP-8.6 samples at the concentrations of 0.05 and 0.25 mg/mL resulted in significantly higher tail DNA% (Fig. S8a). In the H9C2 cell line, the tail DNA% were significantly different from 0.5 mg/mL and 0.1 mg/mL in the TE-NEP-10.6 and TE-NEP-8.6 samples, respectively (Fig. S8b). The amount of tail DNA induced by TE-NEP-10.6 at all treatment concentrations was not different from the control group. In TE-NEP-8.6 samples, there was a significant difference in tail DNA% above 0.1 mg/mL. Treatment of samples at high concentrations (> 1 mg/mL) caused detachment of cells during the washing process, making the cell collection difficult. Therefore, accurate experimental results could not be obtained in the treatment group with high concentration. hCPC was used as a representative of human-derived primary cells and showed more than 35% tail DNA% at all treatment concentrations, which is considered to be slightly toxic to the nanoemulsion compared to the control group.

3. Supplementary Figure

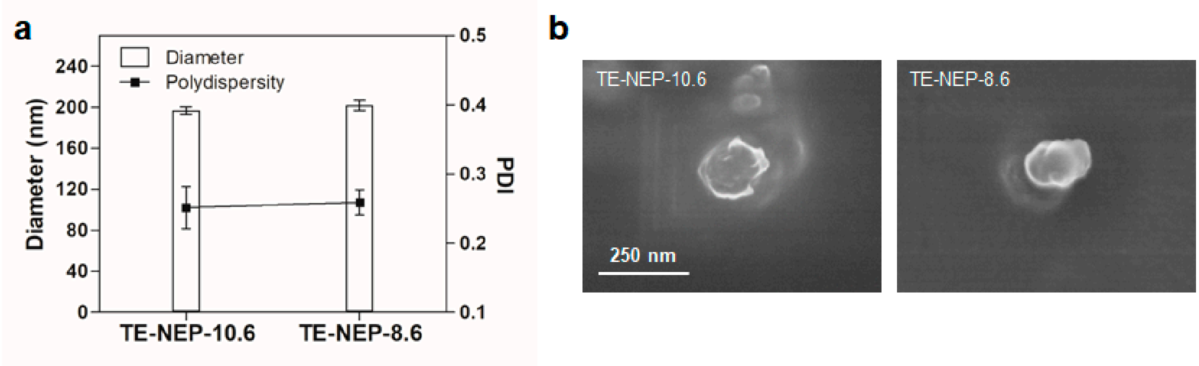

Figure S1. Characterization of two types of nanoemulsions (TE-NEP-10.6 and TE-NEP-8.6). (a) The average diameter size (bars) and polydispersity index (line) of the nanoemulsion. (b) FE-SEM microphotographs of nanoemulsion.

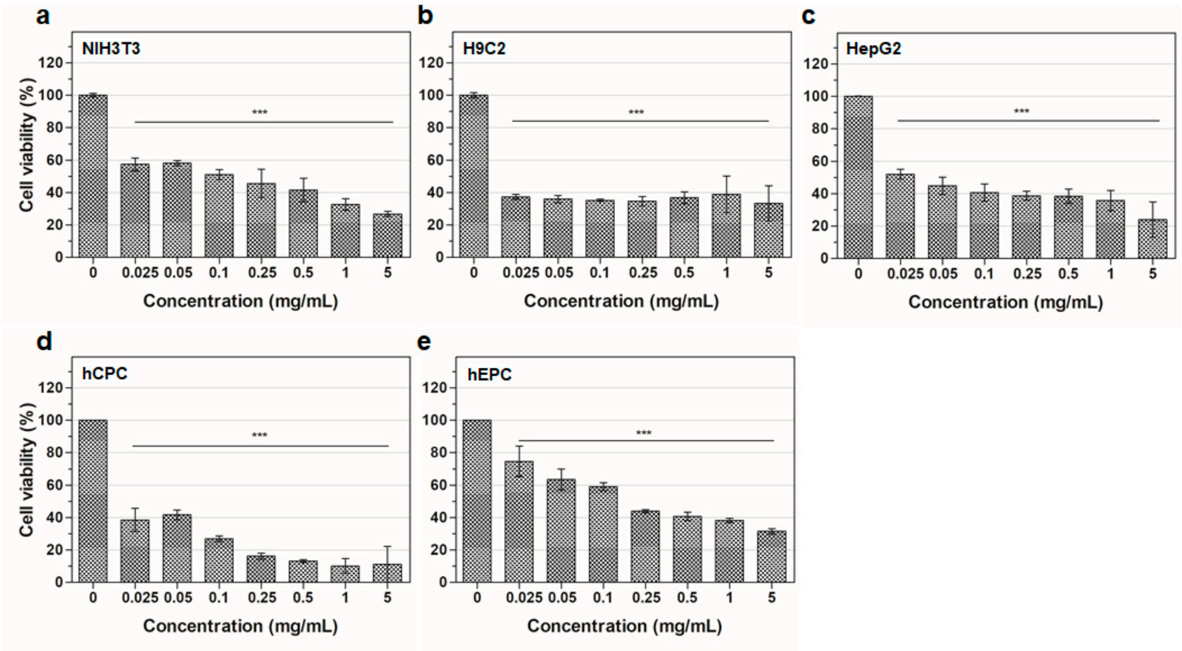

Figure S2. Positive control results of the MTT assay for (a) NIH3T3, (b) H9C2, (c) HepG2, (d) hCPC, and (e) hEPC after the cells were treated with single-wall carbon nanotube (SWCNT) at various concentrations (0.025, 0.05, 0.1, 0.25, 0.5, 1 and 5 mg/mL).

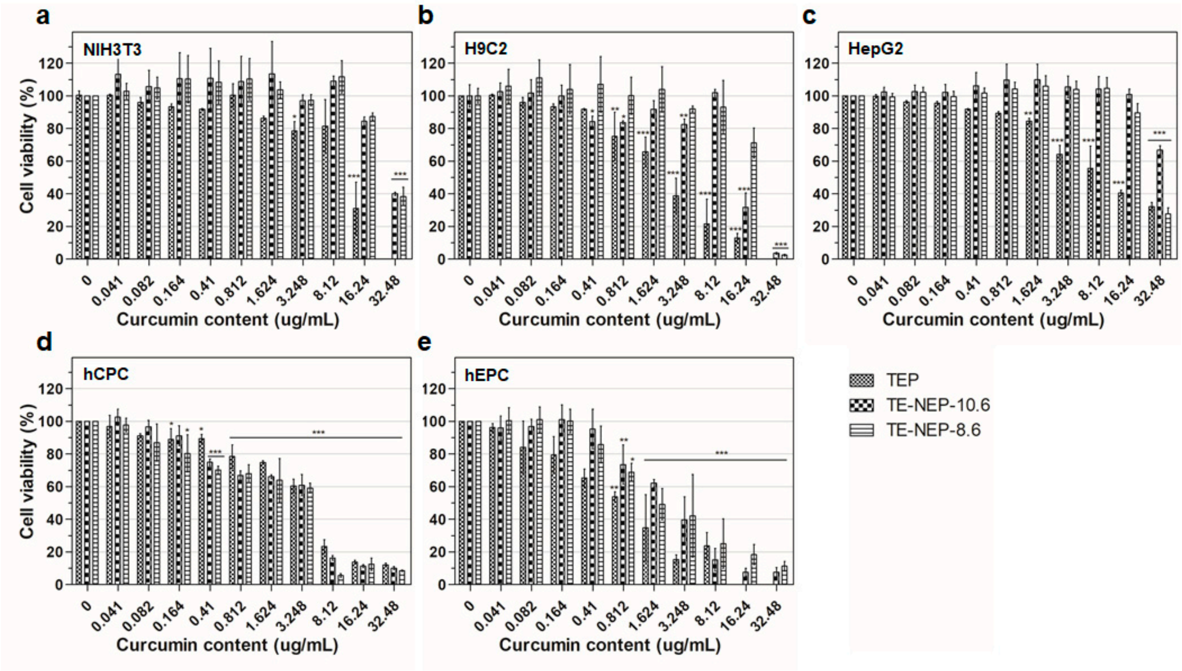

Figure S3. Cell survival rates according to the curcumin content of the sample. (a) NIH3T3, (b) H9C3, (c) HepG2, (d) hCPC, and (e) hEPC were incubated with samples (TEP, TE-NEP-10.6, and TE-NEP-8.6) based on curcumin concentrations (0.041, 0.082, 0.164, 0.41, 0.812, 1.624, 3.248, 8.12, 16.24 and 32.48 μg/mL) for 24 h. Experiments were repeated 3 times independently. $^*p < 0.05$, compared to the control.

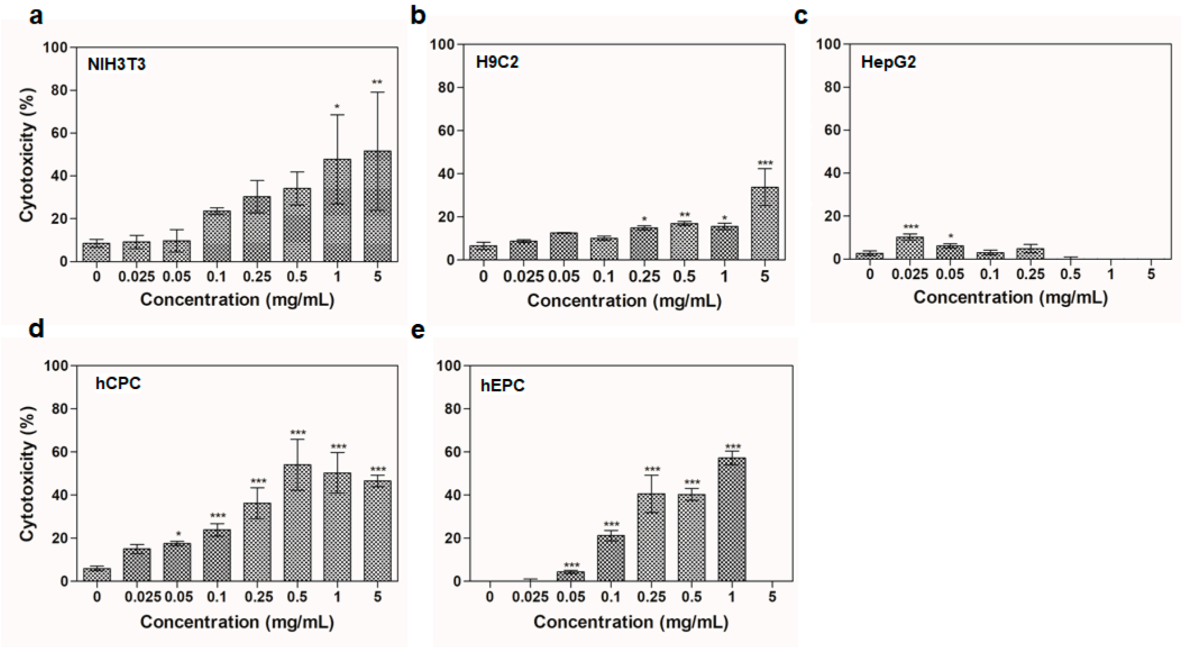

Figure S4. Positive control results of the LDH assay for (a) NIH3T3, (b) H9C2, (c) HepG2, (d) hCPC, and (e) hEPC after the cells were treated with swCNT at various concentrations (0.025, 0.05, 0.1, 0.25, 0.5, 1 and 5 mg/mL).

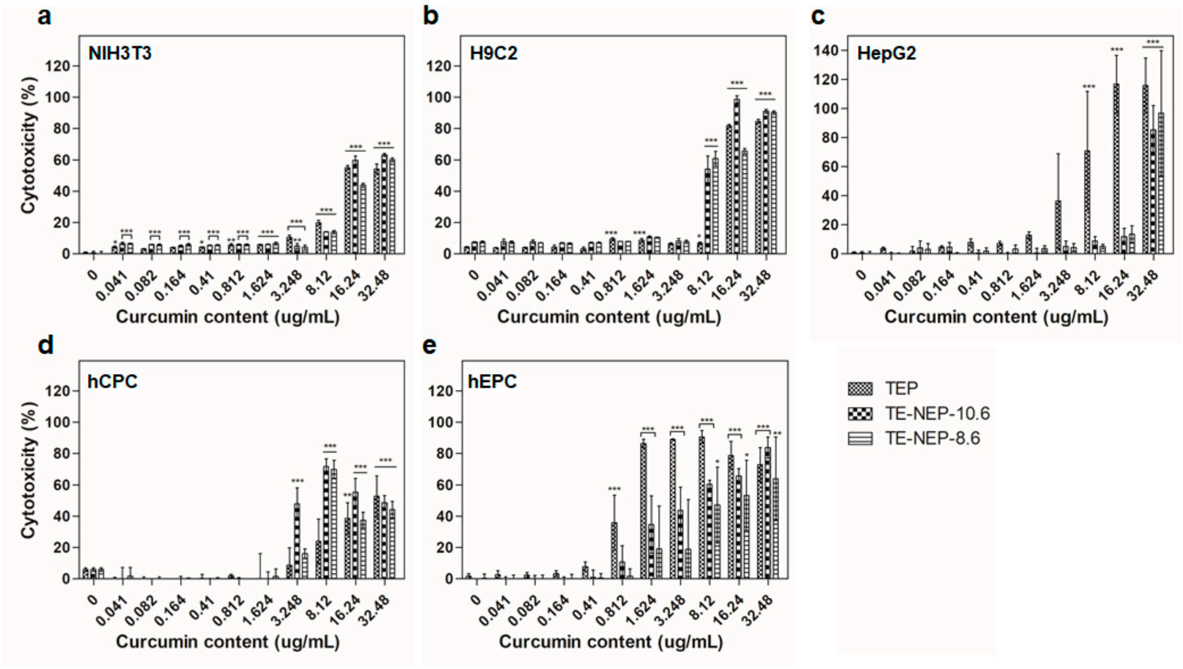

Figure S5. Cytotoxic effects of various curcumin concentrations (0, 0.041, 0.082, 0.164, 0.41, 0.812, 1.624, 3.248, 8.12, 16.24 and 32.48 μg/mL) on (a) NIH3T3, (b) H9C3, (c) HepG2, (d) hCPC, and (e) hEPC. Cell death was measured using the LDH-release assay. Experiments were repeated 3 times independently. *p < 0.05, compared to the control.

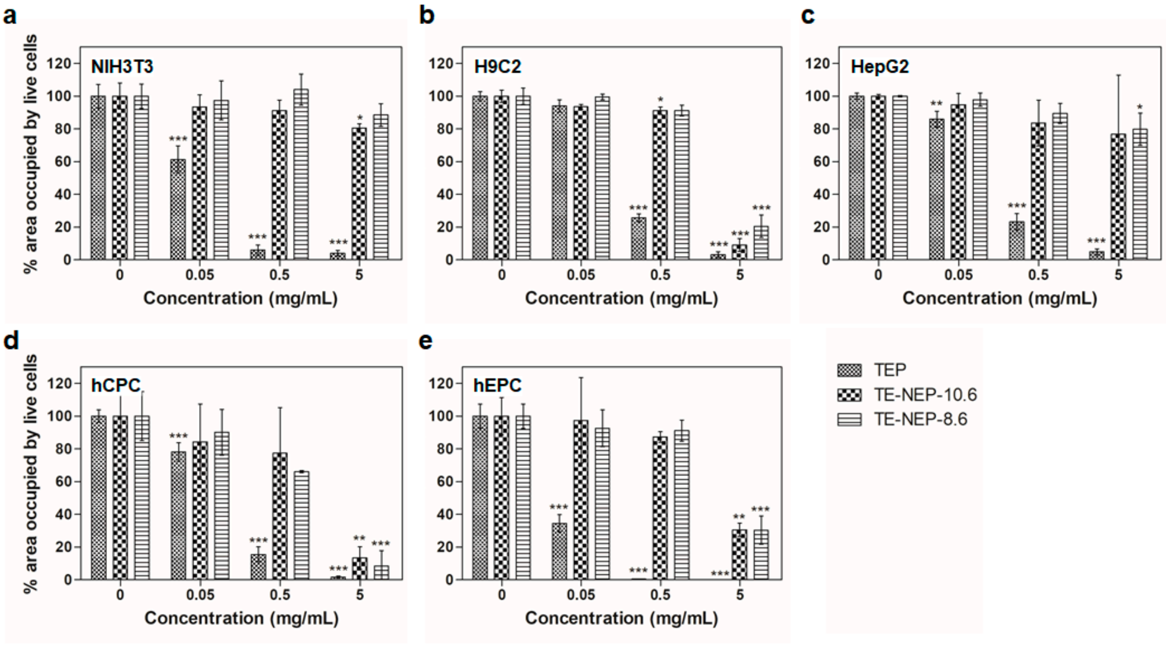

Figure S6. Quantification of the area occupied by live cells through live/dead staining of (a) NIH3T3, (b) H9C3, (c) HepG2, (d) hCPC, and (e) hEPC. The results represent the mean values from 3 independent samples with error bars (standard deviation). The samples were normalized against the untreated group.

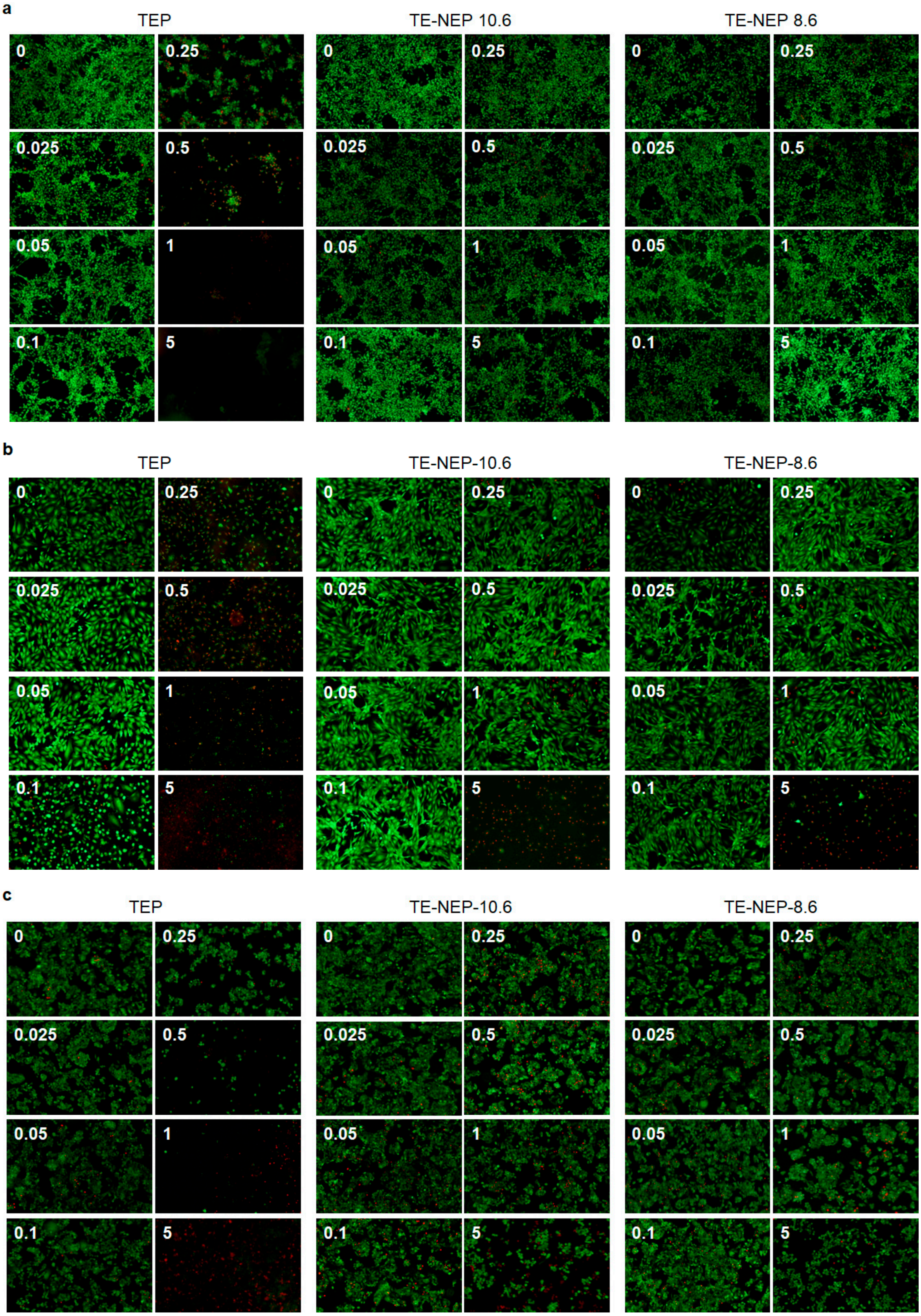
a
TEP
TE-NEP 10.6
TE-NEP 8.6
0
0.25
0.025
0.5
0.05
1
0.1
5
b
TEP
TE-NEP-10.6
TE-NEP-8.6
0
0.25
0.025
0.5
0.05
1
0.1
5
c
TEP
TE-NEP-10.6
TE-NEP-8.6
0
0.25
0.025
0.5
0.05
1
0.1
5

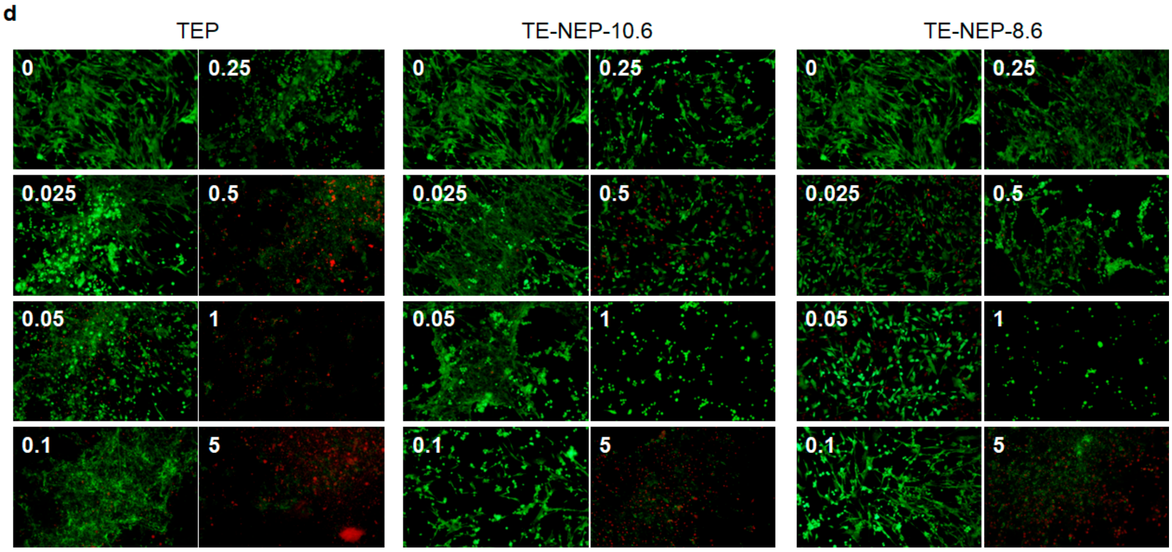

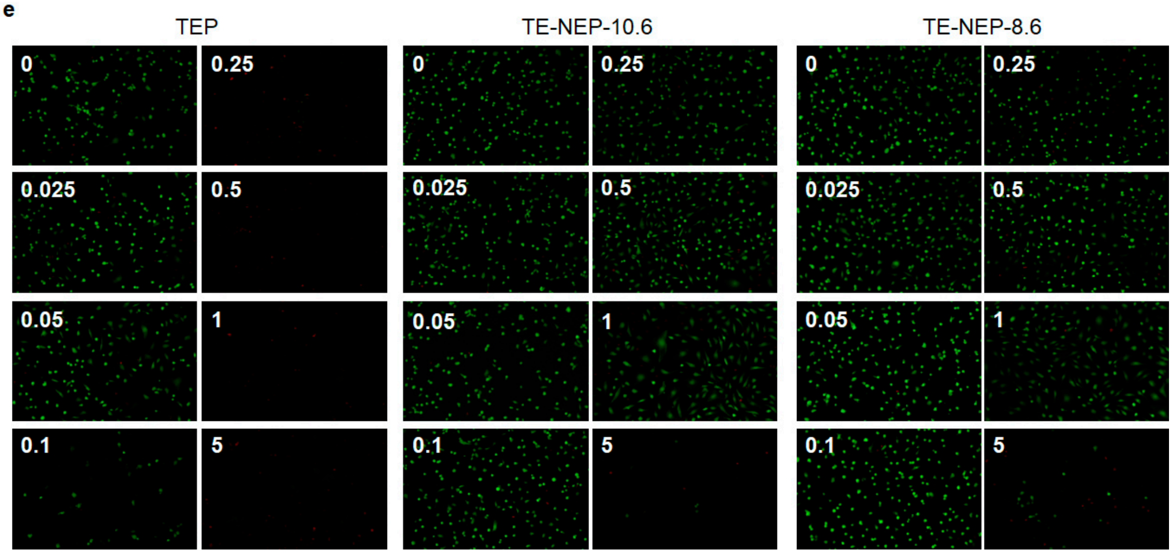

Figure S7. Live/dead fluorescent images for all experimental concentrations (0, 0.025, 0.05, 0.1, 0.25, 0.5, 1 and 5 mg/mL). (a) NIH3T3, (b) H9C3, (c) HepG2, (d) hCPC, and (e) hEPC were stained at 24 hours after the treatment with TEP, TE-NEP-10.6 and TE-NEP-8.6. Live cells were stained with calcein AM (green) and dead cells were stained with ethidium homodimer (red).

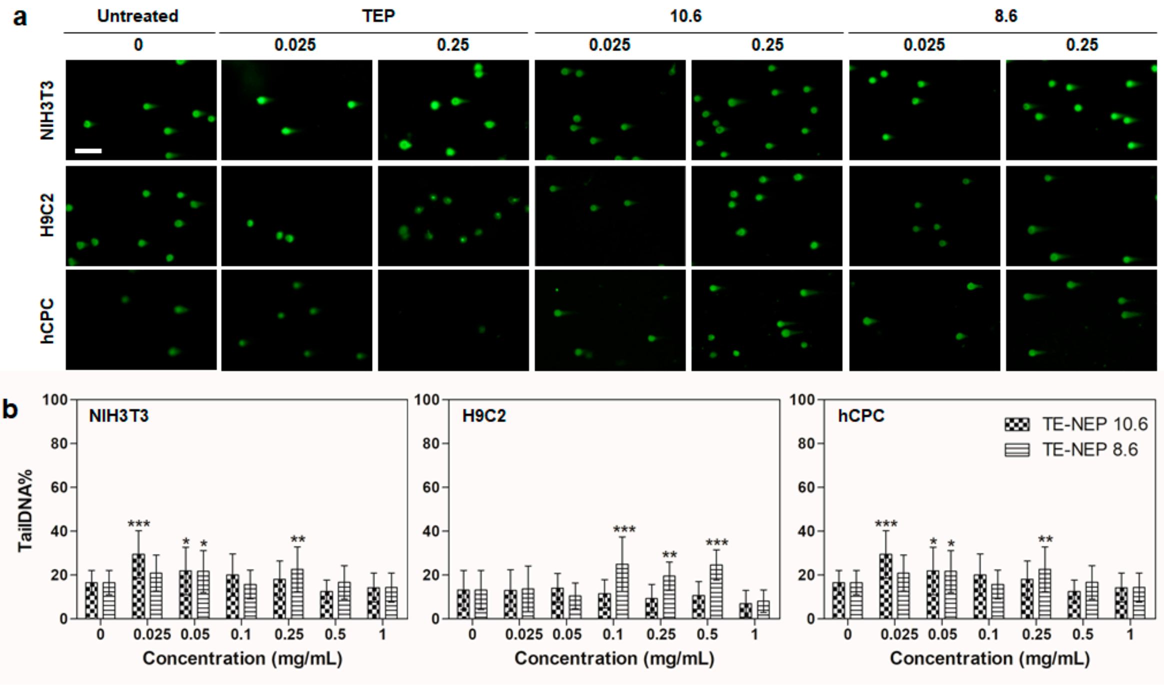

Figure S8. Comet assay results obtained from exposure of 0.025, 0.05, 0.1, 0.25, 0.5 and 1 mg/mL concentrations of nanoemulsion to NIH3T3, H9C2 and hCPC. (a) Representative fluorescence image of Comet Assay. (b) Quantification of DNA damage. scale bar = 100 µm

1. Courilleau, C., et al., *The chromatin remodeler p400 ATPase facilitates Rad51-mediated repair of DNA double-strand breaks.* The Journal of Cell Biology, 2012. **199**(7): p. 1067-1081.
